# Supplementary material for: Readout of a solid state spin ensemble at the projection noise limit
Source: Nat Commun. 2026 May 4;17:4028. doi: 10.1038/s41467-026-72721-0 (PMC13139596; doi:10.1038/s41467-026-72721-0)
Supplement: Supplementary file 1 — Supplementary Information [file 41467_2026_72721_MOESM1_ESM.pdf]

Supplementary Information for ”Readout of a solid state spin ensemble at the projection noise limit”

Rouven Maier[1,2], Cheng-I Ho[1,3], Andrej Denisenko[1], Marina Davydova[4], Peter Knittel[4], Jörg Wrachtrup[1,2,3], and Vadim Vorobyov*[1,3]

[1]3rd Institute of Physics, University of Stuttgart, 70569 Stuttgart, Germany.
[2]Max Planck Institute for Solid State Research, 70569 Stuttgart, Germany.
[3]Center for Integrated Quantum Science and Technology (IQST), 70569 Stuttgart, Germany.
[4]Fraunhofer Institute for Applied Solid State Physics (IAF), 79108 Freiburg, Germany

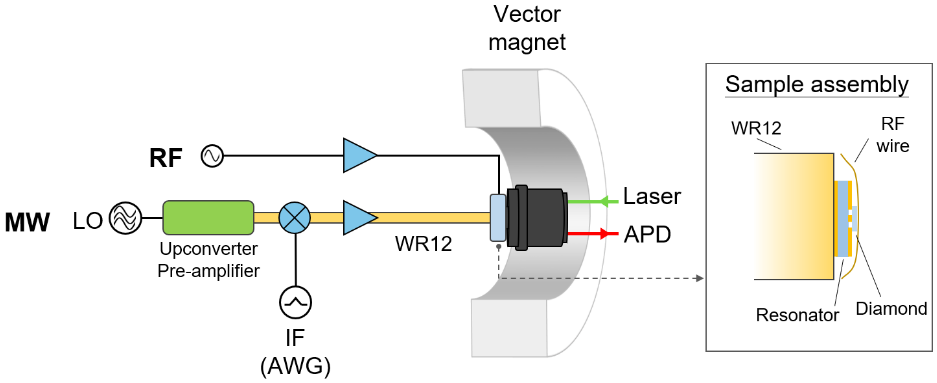

Supplementary Figure 1: **Experimental Setup.** The experiments are performed on a confocal, room-temperature, ensemble-NV setup. The NV-containing diamond is placed inside a superconducting magnet (2.7 T) and optically addressed with a green laser, focused through a high-NA objective. Red fluorescence is collected and detected using an avalanche photodiode (APD). Microwave (MW) pulses at 73 GHz are generated by upconverting the local oscillator (LO) signal six times and mixing it with pulsed MW from the arbitrary waveform generator (AWG). RF control of the nuclear spins is applied to the diamond via a copper wire on its backside. Inset: detailed view of the diamond and resonator.

Supplementary Note 1: Experimental setup

A schematic of the experimental setup is provided in Supplementary Fig. 1. Optical excitation of the NV center is achieved with a pulsed $532\,\mathrm{nm}$ laser gated by a TTL signal. The beam is focused onto the diamond using an oil-immersion objective (Olympus UPlanSApo 60x, NA = 1.35, working distance 300 μm). Fluorescence in the red spectral range is collected through the same objective, passed through a $50\,\mu\mathrm{m}$ pinhole and a 650 nm long-pass filter, and subsequently detected by an avalanche photodiode (APD) connected to a TimeTagger (Swabian

*v.vorobyov@pi3.uni-stuttgart.de

Instruments) for photon counting. The diamond is glued to the resonator, and the combined assembly is mounted onto a WR12 rectangular waveguide. For nuclear spin control a copper wire is positioned on the backside of the diamond. The assembly is centered in the room-temperature bore of a superconducting vector magnet (Scientific Magnetics) at $2.7\,\mathrm{T}$.

Microwave pulses at $\sim$ $73\,\mathrm{GHz}$ are generated by mixing a continuous-wave carrier with pulsed modulation. Specifically, a carrier frequency (Anritsu MG3697C) is upconverted six times, pre-amplified (S12MS, OML Inc.), and mixed (SAGE-SFB-12-E2) with the pulsed output of an arbitrary waveform generator (AWG, Keysight M9505A; frequency range 100–1000 MHz). The resulting microwave signal is further amplified (SAGE-SBP-7137633223-1212-E1 and SAGE-AMP-12-02540) before being coupled to the resonator via the WR12 waveguide. RF control of the nitrogen spins is provided by the same AWG and subsequently amplified (AR150A250). The resonator is fabricated from a double-side polished sapphire substrate (α-Al2O3 (0001), $280\,\mu\mathrm{m}$ thick, commercially available). The resonator structure is realized by copper plating, photolithography, electroplating, reactive-ion etching, and final laser cutting. For further details regarding the resonator design, see [1]. Our MW setup allows Rabi frequencies up to $\sim$ $5\,\mathrm{MHz}$. For the nuclear spin selective MW pulses, the power was reduced to achieve π-pulse durations of $\sim 1.3\mu s$.

The standard Hamiltonian of the electron-nitrogen spin system is given by:

$$\hat{H} = \underbrace{D\hat{S}_z^2 - \gamma_e \hat{S}_z B_z}_{\text{Electron}} + \underbrace{A_{\parallel}\hat{S}_z\hat{I}_z}_{\text{HFS}} + \underbrace{Q\hat{I}_z^2 - \gamma_N \hat{I}_z B_z}_{\text{Nuclear}}\,, \tag{1}$$

where $\gamma_e = -28\,025\,\mathrm{MHz/T}$ and $\gamma_N = 3.077\,\mathrm{MHz/T}$ are the electron and nuclear gyromagnetic ratios, $D = 2870\,\mathrm{MHz}$ is the zero field splitting, $A_{\parallel} = -2.16\,\mathrm{MHz}$ is the hyperfine splitting (HFS), $Q = -4.95\,\mathrm{MHz}$ is the quadrupolar splitting and $B_z = 2.73\,\mathrm{T}$ is the appllied magnetic field. The energy levels and the spin selective transitions are visualized in Supplementary Fig. 2. The electron transitions are used to probe the nuclear spin state in the main readout protocol, while the nuclear transitions are used to initialize the nitrogen spin state (see Fig. 3 a of the main text).

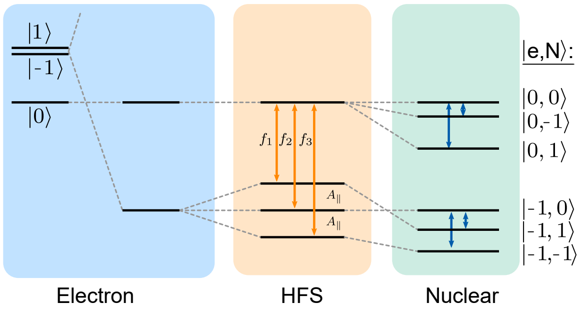

Supplementary Figure 2: **Energy level scheme.** The spin-selective electron (nuclear) transitions are indicated by orange (blue) arrows.

The diamond used in this study was prepared by homoepitaxial growth on a 1.5x1.5 mm^2 (111) IIa HPHT substrate (FSBI TISNCM) with a miscut angle of $1.7°$. Before overgrowth, the substrate was chemically cleaned in nitric acid at $250\,°\mathrm{C}$ and subsurface damage from polishing was removed employing an oxygen ICP plasma etch to remove approx. 2-3 μm of the upmost layer. Overgrowth was carried out employing a custom-build diamond CVD reactor with an ellipsoidal resonator for sharp layer interfaces [2]. Low methane process conditions were chosen to enable the growth of aligned NV centers in a step-flow growth mode [3]. A layer stack consisting of an approx. $1\,\mu\mathrm{m}$ thick intrinsic buffer and the nitrogen doped layer with $277\,\mathrm{nm}$ was grown. Both layers employed $0.5\,\%$ CH_4 in H_2 and growth was carried out at around $850\,°\mathrm{C}$. The ^{14}N-doped layer was grown using isotopically purified $^{12}CH_4$ and nitrogen at an N/C ratio of $40\,000\,\mathrm{ppm}$. From secondary ion mass spectrometry (SIMS), a nitrogen concentration of approx. $2.4 \times 10^{18}\,\mathrm{N/cm^3}$, i.e., $13.6\,\mathrm{ppm}$ was obtained. This corresponds to a doping efficiency of around $0.03\,\%$ as expected from literature [4].

ODMR characterization of the diamond demonstrated a Rabi contrast of up to $30\,\%$ as expected for preferentially aligned NV centers [5]. Typical decay times of the NVs are $T_2^* \sim 0.9\,\mu\text{s}$, $T_2^{\text{Hahn}} \sim 15\,\mu\text{s}$ and $T_1 \sim 3.4\,\text{ms}$. The spin-dephasing times obtained at $2.7\,\text{T}$ correlate to 11 ppm of nitrogen contributing to the spin bath when applying the model described in literature [6], which is in good agreement with the measured concentration from SIMS.

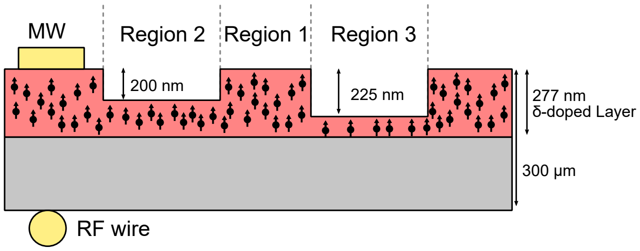

Supplementary Figure 3: **Visualization of the diamond sample.** The overgrown δ-doped layer contains the ^{14}NV centers. Three different NV concentrations in the confocal spot are prepared by etching of the diamond surface. The MW resonator for electron spin manipulation is located on top of the diamond, while the RF wire for nuclear spin manipulation is located at the back.

Supplementary Note 2: Time averaged spin distribution

To beat the photon shot noise in the readout of nitrogen spin states $\tilde{J}_z$, the number of repetitive readouts m (or the readout time T) has to be increased. The main limiting factor here, are small disturbances to the $\tilde{J}_z$ induced by the readout, leading to an effective decay constant T_1. Therefore, the readout results in an effective average over the statistical decay process, yielding $\langle \tilde{J}_z \rangle_T$. The variance of this readout over multiple measurements is given by

$$\text{Var}(\langle \tilde{J}_z \rangle_T) = \left\langle \langle \tilde{J}_z \rangle_T^2 \right\rangle - \left\langle \langle \tilde{J}_z \rangle_T \right\rangle^2 \tag{2}$$

(3)

For now, a single two level $I = 1/2$ system with thermal polarization (i.e. $\left\langle \langle \tilde{J}_z \rangle_T \right\rangle^2 = 0$) is assumed. Thus

$$\begin{aligned}
\mathrm{Var}(\langle \tilde{J}_z \rangle_T) &= \left\langle \left(\frac{1}{T} \int_0^T \tilde{J}_z(t) \mathrm{d}t \right)^2 \right\rangle && (4) \\
&= \left\langle \frac{1}{T^2} \int_0^T \int_0^T \tilde{J}_z(t_1) \tilde{J}_z(t_2) dt_1 dt_2 \right\rangle && (5) \\
&= \frac{1}{T^2} \int_0^T \int_0^T \left\langle \tilde{J}_z(t_1) \tilde{J}_z(t_2) \right\rangle dt_1 dt_2 && (6) \\
&= \frac{2}{T^2} \int_0^T \int_0^{t_2} \left\langle \tilde{J}_z(t_1) \tilde{J}_z(t_2) \right\rangle dt_1 dt_2 && (7) \\
&= \frac{2}{T^2} \int_0^T \int_0^{t_2} \left\langle \tilde{J}_z(t_2 - t_1) \tilde{J}_z(0) \right\rangle dt_1 dt_2 && (8) \\
&= \frac{2}{T^2} \int_0^T \int_0^{t_2} \left\langle \tilde{J}_z(\tau) \tilde{J}_z(0) \right\rangle d\tau dt_2 && (9) \\
&= \frac{2}{T^2} \int_0^T \int_\tau^T \left\langle \tilde{J}_z(\tau) \tilde{J}_z(0) \right\rangle dt_2 d\tau && (10) \\
&= \frac{2}{T^2} \int_0^T (T - \tau) \left\langle \tilde{J}_z(\tau) \tilde{J}_z(0) \right\rangle d\tau && (11)
\end{aligned}$$

The correlation function of the spin state $\tilde{J}_z(t)$ is assumed to be stationary and is given by

$$C(\tau) = \langle \tilde{J}_z(\tau) \tilde{J}_z(0) \rangle = \sigma_0^2 e^{-|\tau|/T_1} \quad (12)$$

where σ_0^2 is the base variance of the readout without decay. Inserting Eq. 12 into Eq. 11 yields

$$\begin{aligned}
\mathrm{Var}(\langle \tilde{J}_z \rangle_T) &= \frac{2}{T^2} \int_0^T (T - \tau) \sigma_0^2 e^{-\tau/T_1} d\tau && (13) \\
&= \frac{2\sigma_0^2 T_1^2}{T^2} \left(\frac{T}{T_1} + e^{-T/T_1} - 1 \right) . && (14)
\end{aligned}$$

The final expression for the standard deviation of the spin readout $\sigma_{\tilde{J}_z}$ is given as

$$\begin{aligned}
\sigma_{\tilde{J}_z} &= \sqrt{\mathrm{Var}(\langle \tilde{J}_z \rangle_T)} && (15) \\
&= \sigma_0 \sqrt{\frac{2T_1^2}{T^2} \left(\frac{T}{T_1} + e^{-T/T_1} - 1 \right)} . && (16)
\end{aligned}$$

When reading out the thermal spin state of a spin ensemble, σ_0 is given by the spin projection noise $\sigma_0 = \frac{1}{N_{\mathrm{NV}}} \sqrt{N_{\mathrm{NV}} \frac{I(I+1)}{3}}$. Experimentally, our spin state readout is limited to the two active NV levels. Thus, the three spin states of the ^{14}N nuclear spins ($I = 1$) are mapped onto the electron spin via $|1\rangle_{\mathrm{N}} \to |1\rangle_e, |0\rangle_{\mathrm{N}} \to |0\rangle_e, |-1\rangle_{\mathrm{N}} \to |0\rangle_e$, effectively binning the spin 1 system into a spin 1/2 system. This leads to an effective narrowing of the observed spin distribution, as well as a shift of the mean value. This reduces the observed width by an additional factor of $\sqrt{\frac{1}{3}}$, resulting in the final analytical expression

$$\sigma_{\tilde{J}_z} = \sqrt{\frac{I(I+1)}{3}} \sqrt{\frac{1}{N_{\mathrm{NV}}}} \sqrt{\frac{1}{3}} \sqrt{\frac{2T_1^2}{T^2} \left(\frac{T}{T_1} + e^{-T/T_1} - 1 \right)} . \quad (17)$$

Supplementary Table 1: Expectation value and projection noise of the spin distribution during the readout. Both expressions are given for the spin distribution at time T, as well as the time-averaged spin distribution between 0 and T. These equations are used to model the data of Supplementary Fig. 4.

Averaging type	Expectation value $\langle\cdots\rangle$	Projection noise σ
Non-averaged	pIe^{-T/T_1}	$\sigma_0\sqrt{1-(pe^{-T/T1})^2}$
Time-averaged	$pI(1-\frac{T_1}{T}e^{-T/T1})$	$\sigma_0\sqrt{\frac{2T_1^2}{T^2}(\frac{T}{T_1}+e^{-T/T1}-1)-(p\frac{T_1}{T}(1-e^{-T/T1}))^2}$

This expression captures the spin projection noise (i.e. statistical polarization) of a thermal spin ensemble of spin I particles, as detected in Fig. 2 of the main text. Ideally, a two level quantum memory like the $I = 1/2$ ^{15}N would be used in this experiment, rendering binning unnecessary and improving the efficiency of the nuclear spin polarization. However, the observed spin projection noise (Eq. 17) would almost remain the same as $\sqrt{\frac{1(1+1)}{3}}\sqrt{\frac{1}{3}} \approx \sqrt{\frac{0.5(0.5+1)}{3}}$. For completeness, here we also provide the expressions for expectation value and the standard deviation of the non-time averaged distributions $\tilde{J}_z$, as well as the time-averaged distributions $\langle\tilde{J}_z\rangle_T$ with respect to their polarization p in Supplementary Tab. 1. A Monte-Carlo simulation of the ensemble readout of $N_{\mathrm{NV}} = 100$ spin-1/2 nitrogen spins was performed to visualize the effects of the readout on the underlying spin distributions (Supplementary Fig. 4). Due to the readout-induced decay of the spin polarization, a clear distinction between the projection noise of thermal and polarized spin distribution is getting increasingly difficult, as the readout time T approaches T_1 (orange curves in Supplementary Fig. 4 b). Thus, in all our experiments of the main text the readout duration was fixed to a time significantly shorter than the respective T_1.

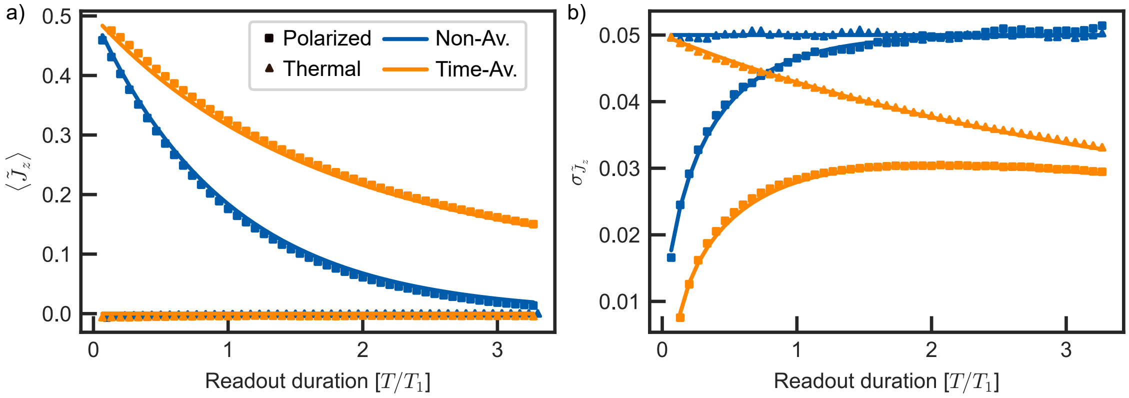

Supplementary Figure 4: **Simulation of the effect of T_1-decay during the readout on expectation value (panel a) and standard deviation (panel b) of the detected spin distribution.** The properties of the spin distribution of $N_{\mathrm{NV}} = 100$ at time T are visualized in blue, while the distribution obtained by averaging the spin distributions between 0 and T are visualized in orange. The properties of a thermal state (triangles) and a polarized state (squares) are shown.

Supplementary Note 3: Spin projection noise transition

In the realization of this experiment, the distribution of $\tilde{J}_z$ is optically recorded by detecting the emitted photons of an ensemble of NVs using an avalanche photodiode (APD). The distribution of detected photons $b-a$ is governed

by the variance sum of the photon shot noise $\sigma_n = \sqrt{a+b} = \sqrt{1-\frac{c}{2}}\sqrt{2n}$ and the spin distribution $\sigma_{\tilde{J}_z}$ via

$$\sigma^2 = \sigma_n^2 + (2nc\sigma_{\tilde{J}_z})^2 \ , \tag{18}$$

where c is the optical contrast of the NV readout. Due to the high photon emission rate of the NV ensembles of up to 6000 kilocounts per second, our APD is no longer working in the linear regime, leading to an artificial reduction of the detected photon width according to

$$\sigma^2 = k^2 \left(\sigma_n^2 + (2nc\sigma_{\tilde{J}_z})^2\right) \ . \tag{19}$$

The reduction factor k can be easily determined by calibrating the effective photon shot noise in the readout of the reference measurements of the baseline counts (Supplementary Fig 5). For this, the relative width of the Skellam distribution obtained by subtracting both reference readouts r_1 and r_2 is determined, and compared to the theoretical photon shot noise of $\sigma_n/n = k\sqrt{2/n}$. The extracted data show ideal photon shot noise limited behavior as $\sigma_n \propto \sqrt{n}$, showing that our distribution width is not artificially increased, e.g. through slow laser drifts or other noise sources. As such, the photon shot noise obtained from the reference measurements is also visualized as the photon shot noise data in Fig. 2 d) in the main text. Thus, any increase in the detected width of the measurement readout $b-a$ can be attributed to contributions originating from the underlying spin distribution.

The model used to fit the measurement data in Fig. 2 d) in the main text and Supplementary Fig. 6 is given by Eq. 19 and Eq. 17, with N_{NV}, T_1 and k as free fit parameters. The fit results for three regions with different NV concentrations are shown in Supplementary Fig. 6 and the fit parameters are given in Supplementary Tab. 2. Instead of the time of the readout T and the time decay T_1, the data is fitted as a function of the number of photons n and its decay n_{T_1}. The extracted values of the reduction factor k closely match those obtained in the calibration of baseline photon shot noise, further validating the approach. Over the duration of the measurement, the T_1 decay of the nitrogen spin state does not yet significantly affect the observed distribution, leading to a large uncertainty of T_1 from the fit. The extracted number of NVs of 170(10), 31(3) and 14(1) for region 1, 2 and 3, respectively, decrease with decreasing height of the NV layer, as expected. Typically, optical readout of NV centers is limited by charge state infidelities, as some NV centers are initialized into the optically inactive NV^0 charge state, instead of NV^-. It has to be noted that in our approach the NV centers are repeatedly re-initialized over the duration of a single readout loop, so that eventually all NVs contribute to the acquired signal. As a result this offers a direct method to directly determine the actual number of NV centers in the confocal spot, instead of only the fraction of NV centers in the negative charge state.

Robust validation of our method to determine the total number of NV centers in the detection volume is not trivial, as no other such method exists to this day. One approach is to compare the extracted numbers with the observed steady state fluorescence emission rate, as more NV centers typically should emit more photons. However, this approach has to be treated very carefully, as the real emission rate does not necessarily increase linearly with the number NVs in the confocal volume, as the emission rate depends on complicated steady state charge dynamics in the beam profile of the excitation laser. In our case, the extracted number NVs follows a linear relation to the photon emission rate r under green excitation (Supplementary Fig. 6 c). The linear fit function is given by $r = n'_1 N_{\mathrm{NV}} + r_0$, where $r_0 = 270(190)\,\mathrm{kcps}$ is the background fluorescence and $n'_1 = 32(2)\,\mathrm{kcps/NV}$ is the emission rate per NV.

A second option to roughly estimate the number of NV centers in the detection volume is by geometric considerations. Assuming a confocal limited spot with a diameter of $d_{\mathrm{beam}} = \frac{\lambda_0}{0.84\mathrm{NA}}$ (Gaussian beam profile with 1σ radius), where $\lambda_0 = 532\,\mathrm{nm}$ is the wavelength of the laser and NA the numerical aperture of the objective, the number of nitrogen atoms N_{N} in the $277\,\mathrm{nm}$ thick NV layer can be estimated by using the nitrogen density $[\mathrm{N}] = 11\,\mathrm{ppm}$ obtained by secondary ion mass spectroscopy (SIMS) and comparison to another calibration sample, yielding $N_{\mathrm{N}} \approx 36000$. The rate of NV centers formed for each Nitrogen atom in this non-annealed sample is assumed on the order of $0.3\,\%$ based on previous calibrations, yielding an estimate for the number of NVs in the confocal volume on the order of $N_{\mathrm{NV}} \approx 140$. This number is close the extracted number of $N_{\mathrm{NV}} = 170(10)$, but it is again only a rough estimate as for example the NV conversion rate cannot be determined exactly and additional NVs from outside the 1σ region might contribute to the observed signal. For the thinner, etched regions (Region

2 and 3) this calculation of the average expected number of NV centers cannot be performed, as in these regions inhomogeneous distribution of the NV centers could clearly be seen in the confocal image.

Nevertheless, as both, the comparison to the photon emission rate, and the expected number of NVs from geometric considerations confirm the extracted number of NV centers from the spin projection noise, we are confident in its validity.

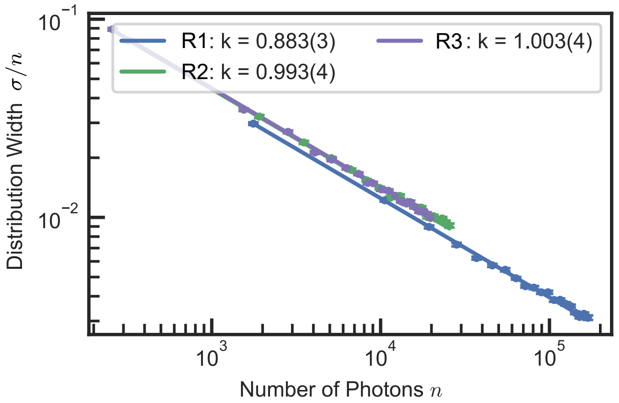

Supplementary Figure 5: **Calibration of the photon shot noise.** Detected distribution width σ/n, when subtracting both reference measurements r_1 and r_2. The fit function is $\sigma/n = k\sqrt{2/n}$.

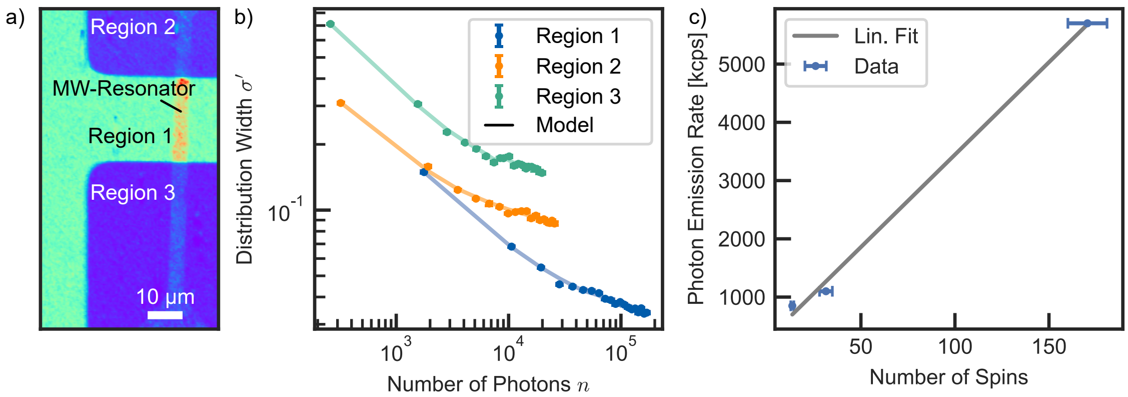

Supplementary Figure 6: **Determination of number of NVs in the confocal spot. a)** Confocal scan image. Three regions with different NV concentrations and the microwave resonator are visible. **b)** transition from the photon shot noise dominated regime to the spin projection noise dominated regime. The fit parameters of the model from Eq. 19 are given in Supplementary Tab. 2. **c)** Linear relation between the extracted number of NVs and the photon emission rate under laser excitation.

Supplementary Note 4: T_1-decay of spin polarization

At a high magnetic field of 2.7 T the nitrogen spin states are stabilized to achieve a quantum-non-demolition measurement condition. However, during the large number of readout repetitions applied in this work small perturbations add up and the spin state shows a characteristic decay induced by perpendicular hyperfine interaction, thus setting an upper limit to the possible number of readouts. For the purpose of this work, we refer to this polarization decay as a T_1 process, even though the decay is much faster than the natural spin lattice relaxation, which

Supplementary Table 2: Fit parameters of projection noise transition fit according to Eq. 19

Region	Emission Rate [kcps]	N_{NV}	n_{T_1} [10^3 counts]	k
1	5700	170(10)	582(469)	0.89(1)
2	1100	31(3)	1600(22092)	0.99(2)
3	850	14(1)	3222(2436)	0.98(1)

can reach minutes at the applied magnetic field. [7] During the duration of the readout T the polarization p decays to the steady-state thermal polarization p_{ss} according to the exponential decay $p = p_0(1-p_{\text{ss}})e^{-T/T_1} + p_{ss}$. Since our readout bins both, $|0\rangle$ and $|-1\rangle$ into the same electron spin state, the polarization decays to a steady state polarization of $p_{\text{ss}} = -\frac{1}{3}$. Averaging of the spin state decay during the full readout duration T yields the observed polarization

$$p_{\text{obs}} = \frac{1}{T}\int_0^T p dT = p_0(1-p_{\text{ss}})\frac{T_1}{T}\left(1 - e^{-T/T_1}\right) + p_{\text{ss}} = p_0(1-p_{\text{ss}})\frac{m_{T_1}}{m}\left(1 - e^{-m/m_{T_1}}\right) + p_{\text{ss}} \ . \tag{20}$$

The final expression is expressed in terms of the readout repetition number m instead of the total readout duration T. The polarization decay under readout for the nitrogen spin states $|1\rangle$ and $|0\rangle$ are shown in Supplementary Fig. 7. The $|0\rangle$ state shows a twice faster decay compared to the $|1\rangle$ state, mainly because of additional relaxation pathways through the spin-allowed transitions into both, $|1\rangle$ and $|-1\rangle$ states. This faster decay rate in $|0\rangle$ is the main reason for the larger spin projection noise during the inversion of the nitrogen rabi drive in Fig. 3 b) of the main text.

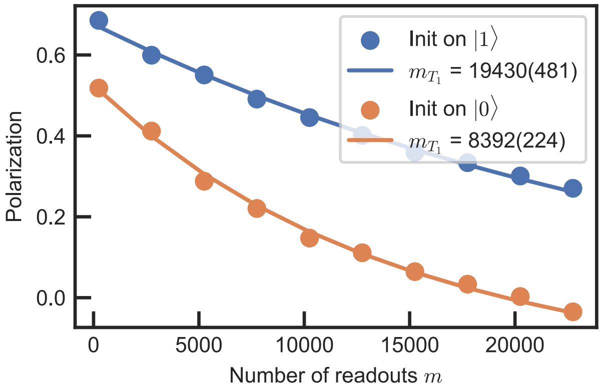

Supplementary Figure 7: **Nitrogen Spin Polarization Decay.** The nitrogen spin level $|1\rangle$ and $|0\rangle$ show a decay to 1/e after $m_{\text{T}_1} = 19430$ and 8392 readouts, respectively. The experimental data is fitted by Eq. 20.

Supplementary Note 5: Noise spectroscopy using dynamical decoupling

When using noise spectroscopy based on dynamical decoupling (DD), the sensor is selectively coupled to the frequency of an oscillating noise source with the signal

$$S = B_{\text{osc}} \cos\left(2\pi f t + \lambda\right) \ , \tag{21}$$

where B_{osc} is the magnetic field amplitude, f is the frequency and λ is the random phase of the signal. By sweeping the inter-pulse delay τ of the decoupling sequence, the sensor spin along the +Y axis acquires a phase [8]

$$\theta = \alpha \text{sinc}((\tau - \tau_0)N_p\tau\pi)\sin(\lambda) = \alpha' \sin(\lambda) \ . \tag{22}$$

Here $\tau_0 = \frac{1}{2f}$ is resonant pulse spacing, N_p is the number of applied π pulses and $\alpha = 2\pi\frac{2}{\pi}B_{\text{osc}}\gamma_e\tau_{\text{sens}}$ is the interaction strength between the electron spin and the oscillating target field during the sensing time τ_{sens} [9]. $\gamma_e = 28.04\,\text{GHz/T}$ is the gyromagnetic ratio of the electron spin. The expectation values along X and Y are given by

$$\langle X\rangle = \langle\sin(\theta)\rangle_\lambda = 0 \;, \tag{23}$$

$$\langle Y\rangle = \langle\cos(\theta)\rangle_\lambda = 0.5J_0(\alpha') \;, \tag{24}$$

where $\langle\rangle_\lambda$ is the average over the random phase λ and J_0 is the zeroth order Bessel function of the first kind. The standard deviation of the X and Y readout are then given by

$$\sigma_x = 0.5\sqrt{\frac{1-J_0(2\alpha')}{2}} \;, \tag{25}$$

$$\sigma_y = 0.5\sqrt{\frac{1+J_0(2\alpha')}{2} - J_0(\alpha')^2} \;. \tag{26}$$

The projection of the spin ensemble along Z remains unaffected by the DD-detection sequence in the unpolarized state ($\langle Z\rangle = 0$, $\sigma_z = \sigma_{\text{thermal}}$). Under our experimental conditions, the observed standard deviation of the distribution is influenced by the remaining photon shot noise via $\sigma' = \sqrt{\left(\frac{\sigma_n}{2nc}\right)^2 + \sigma_{x/y/z}^2}$. For fitting the experimental data in Fig. 4 c) in the main text, an additional scaling parameter k_1 was introduced to account for readout infidelities leading to a reduction in the amplitudes:

$$\sigma' = \sqrt{\left(\frac{\sigma_n}{2nc}\right)^2 + k_1\sigma_{x/y/z}^2} \tag{27}$$

In the experiments depicted in Fig. 4 of the main text, a frequency of $f = 250\,\text{kHz}$ and a signal amplitude of $B_{\text{osc}} = 1.84(7)\,\mu\text{T}$ at the position of the NV center was generated by the AWG and supplied by the same wire as the RF for the nuclear spin drive. A standard XY8 decoupling sequence was applied to detect the signal.

Supplementary Note 6: Magnetic field dependence of noise transition

From an experimental point of view, an important question is, which magnetic field needs to be applied in order to be able to observe the transition from the photon shot noise limited regime to the spin projection noise limited regime. Here, we calculate these numbers for our experimental setup. The spin projection noise as a function of the effective decay time m_{T_1} and the number of readouts m, based on equation 19 is given by:

$$\sigma_{\tilde{J}_z} = \sqrt{\frac{I(I+1)}{3}}\sqrt{\frac{1}{N_{\text{NV}}}}\sqrt{\frac{1}{3}}\sqrt{\frac{2m_{T_1}^2}{m^2}\left(\frac{m}{m_{T_1}} + e^{-m/m_{T_1}} - 1\right)} \;. \;, \tag{28}$$

while the photon shot noise is given by:

$$\sigma_n' = \frac{1}{c\sqrt{2n}}\sqrt{1-\frac{c}{2}} = \frac{1}{c\sqrt{2n_1 m}}\sqrt{1-\frac{c}{2}}\sqrt{\frac{1}{N_{\text{NV}}}} \;, \tag{29}$$

based on equation 1 in the main text, where we used $n = n_1 m N_{\text{NV}}$, with the number of photons per NV per readout n_1. Depending on the effective decay constant m_{T_1}, an intersection between $\sigma_{\tilde{J}_z}$ and σ_n' is possible at the point m' (Supplementary Fig. 8 a). As both expressions show a dependence of $\sqrt{\frac{1}{N_{\text{NV}}}}$, m' does not depend on the size of the ensemble. As the effective decay constant m_{T_1} depends quadratically on the applied magnetic field [10], it is possible to numerically solve $\sigma_{\tilde{J}_z} = \sigma_n'$ for different magnetic fields and extract the necessary number of

readouts necessary to observe the transition (Supplementary Fig. 8 b). In our case, the transition should be barely observable from $0.16\,\mathrm{T}$, but stabilizes around $0.5\,\mathrm{T}$. The parameters used for the calculation are $n_1 = 0.036$, $c = 15\,\%$ and $m_{T_1} = 50000\left(\frac{B}{B_0}\right)^2$, with $B_0 = 2.72\,\mathrm{T}$. However, the necessary magnetic field increases, as the desired ratio between $\sigma_{\tilde{J}_z}$ and σ'_n increases.

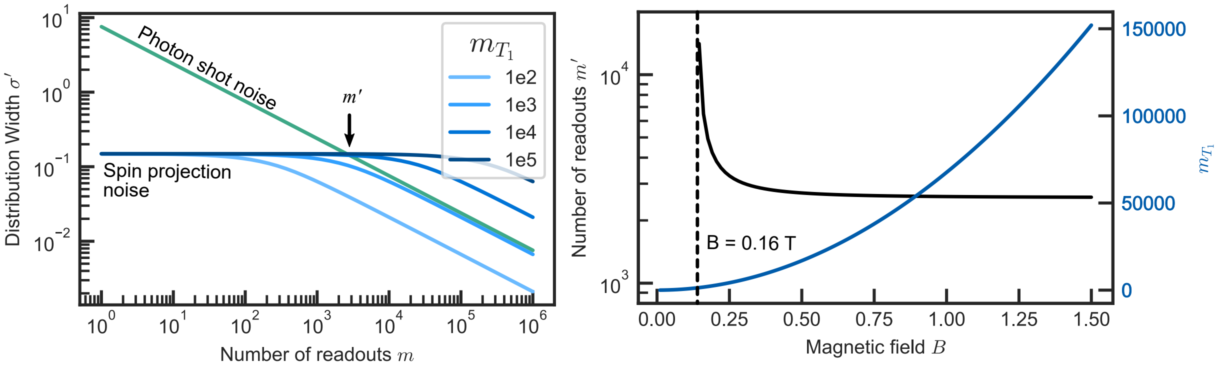

Supplementary Figure 8: **Magnetic field dependence of the crossover from photon shot noise limit to spin projection noise limit.** **a)** Comparison of spin projection noise and photon shot noise for different values of effective m_{T_1}. If m_{T_1} is large enough, an intersection at the point m' can be observed. **b)** Theoretical quadratic behavior of the effective m_{T_1} with increasing magnetic field (blue curve). When m_{T_1} reaches a large enough value at $B \sim 0.16\,\mathrm{T}$, weak crossover from photon shot noise dominated readout to spin projection noise dominated readout becomes possible. The crossover stabilizes for $B > 0.5\,\mathrm{T}$.

Supplementary Note 7: Reconstruction of the Husimi Q-function

In our experiment, the electron spin interacts with coherent RF field with a random phase, leading to a redistribution of the initialized spin state. To reconstruct the Husimi Q-function of the collective spin state, we measure a set of marginal spin distributions $P_S(m_J, \theta, \phi)$ along different quantization axes, defined by the azimuthal and polar angles θ and ϕ, as well as the magnetic quantum number $m_J \in [-J..J]$ along the chosen axis. Namely, these are one projection along the Z-axis ($\theta = 0°$) and nine equally spaced projection axes in the equatorial plane ($\theta = 90°, \phi \in [-90°, 90°]$). Each measured distribution corresponds to the projection of the quantum state aligned with the chosen axis. The obtained histogram data $(b-a)/(2nc)$ is a convolution of the photon shot noise, given by a Skellam distribution, and the underlying spin marginal distribution P_S. For the reconstruction of the spin states, first the deconvolution is achieved using a maximum-likelihood algorithm. In the next step, the obtained distribution was normalized by the obtained borders of the nitrogen Rabi measurement (Fig. 3 a) of the main text), to isolate only active spins (i.e. NV centers with the correct charge and spin state) and remove NV centers where the nitrogen spin was initialized into a state other than $|+1\rangle$, since these spins remain in the polarized state. From these normalized marginal distributions, we employ an inversion algorithm that maps the experimentally acquired probability distributions onto the Husimi Q-function [11], defined as

$$Q(\theta, \phi) = \frac{1}{\pi}\langle\theta, \phi|\rho|\theta, \phi\rangle \ , \tag{30}$$

where $|\theta, \phi\rangle$ are spin-coherent states on the Bloch sphere [12]. This procedure yields a quasiprobabilistic distribution that provides a phase-space representation of the measured state, allowing us to directly visualize its coherence properties, quantum fluctuations and nonclassical features. In practice, the total spin state ρ is expanded as a sum

of $i = 51$ spin coherent states ρ_{coh} and a thermal part ρ_{therm}:

$$\rho = \sum_{k=0}^{i} a_k \rho_{\text{coh}}(\theta_k, \phi_k) + a_{\text{therm}} \rho_{\text{therm}} \ , \tag{31}$$

where a_k, a_{therm} are the respective weights. The choice of potential spin coherent states is physically informed and limited to states equally spaced in a polar angle range of $[-\Delta\phi, \Delta\phi]$ around $\phi = 90°$ for a single θ with equal weights a_k, as expected for a spin system interacting with an oscillating field. A maximum likelihood algorithm solves for the optimum values of $\Delta\phi$, θ and a_{therm} for a given number of spins N_{NV} (here $N_{\text{NV}} = 26$) in the Dicke basis [13]. As shown in Supplementary Fig. 9, the reconstructed spin state ρ reproduces the measured marginal distributions well. From the obtained spin coherent states $\sum_{k=0}^{i} a_k \rho_{\text{coh}}(\theta_k, \phi_k)$ the Husimi Q-function can be easily calculated and is visualized on a sphere. The thermal contribution is a constant offset in the Husimi Q-function and is therefore omitted in the surface plots.

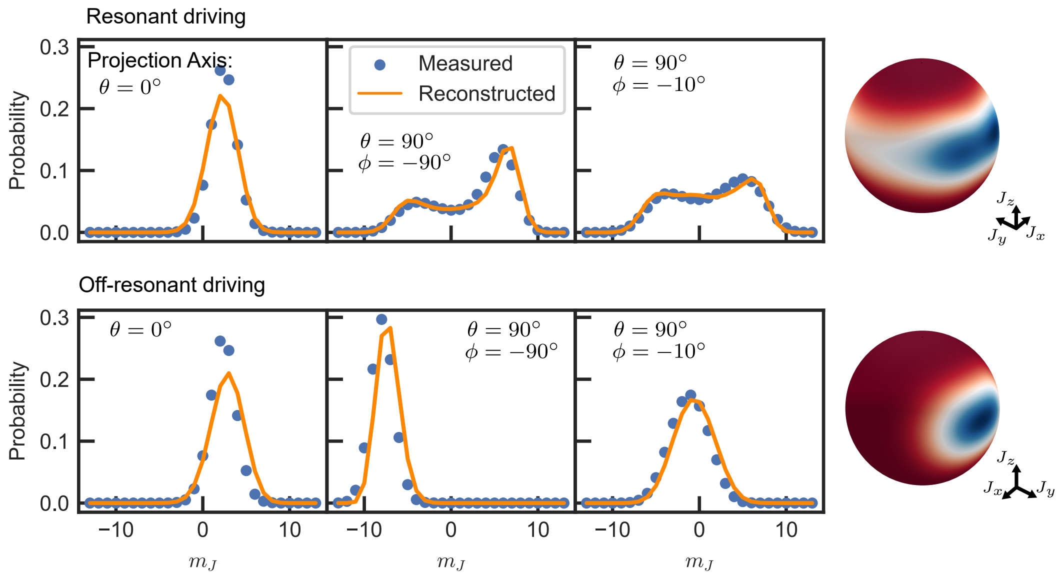

Supplementary Figure 9: **Reconstruction of the spin state.** Comparison of the measured marginal distributions (blue) and the reconstructed marginal distributions (orange) along three different projection axes. The Husimi Q-function of the coherent part of the reconstructed spin state is visualized on a sphere.

Supplementary Note 8: Sensitivity improvement through projection noise limited readout

To investigate the applicability of the repetitive readout of ensemble spin systems to sensing protocols, here we calculate the sensitivity and compare it to the conventional single readout. Generally, the conventional readout scheme is fast but limited to the photon shot noise, while the repetitive readout can reach the spin projection noise at the cost additional experimental overhead for memory spin initialization and increased readout duration. The sensitivity η is defined as the minimal detectable signal δB with a signal-to-noise ratio of 1 during the measurement

time T_{meas} [9]

$$\eta = \delta B \sqrt{T_{\mathrm{meas}}} = \frac{1}{\gamma_e c_{\mathrm{eff}} n \sqrt{\tau_{\mathrm{sens}}}} \sqrt{\frac{\tau_{\mathrm{sens}} + \tau_{\mathrm{other}}}{\tau_{\mathrm{sens}}}} \sigma \tag{32}$$

$$\delta B = \frac{1}{2\pi \gamma_e c_{\mathrm{eff}} n \tau_{\mathrm{sens}}} \sigma \tag{33}$$

$$T_{\mathrm{meas}} = \tau_{\mathrm{sens}} + \tau_{\mathrm{other}} \tag{34}$$

Here slope detection is assumed with a linear sensor response, and σ is the total noise of the readout, $\gamma_e = 28.04\,\mathrm{GHz/T}$ the gyromagnetic ratio of the electron spin, c_{eff} the effective contrast, τ_{sens} the sensing time and τ_{other} experimental overhead (e.g. duration of initialization and readout). In the conventional readout, the noise is given by $\sigma \approx \sqrt{n}$, where $n = n_1$ is the photons detected in a single readout and the effective contrast is equal to the optical contrast of the NV center $c_{\mathrm{eff}} = c$. The experimental time overhead τ_{other} consists only of the duration of a single readout τ_{r}, leading to

$$\eta_{\mathrm{conv}} = \frac{1}{2\pi \gamma_e c n_1 \sqrt{\tau_{\mathrm{sens}}}} \sqrt{\frac{\tau_{\mathrm{sens}} + \tau_{\mathrm{r}}}{\tau_{\mathrm{sens}}}} \sqrt{n_1}\ . \tag{35}$$

In the repetitive readout, a double-sided scheme is applied (i.e. using the photon count difference $b - a$), doubling the effective contrast $c_{\mathrm{eff}} = 2c$, while increasing the photon shot noise by a factor of $\sqrt{2}$. Additionally, the effective contrast is reduced by the decay of the nitrogen spin state during the m readouts with the characteristic decay rate of m_{T_1}, leading to $c_{\mathrm{eff}} = 2c\frac{m}{m_{T_1}}\left(1 - e^{-m/m_{T_1}}\right)$. The noise σ in the readout of a superposition spin state (i.e. maximum spin projection noise) of a spin 1/2 system is given by

$$\sigma = \sqrt{\sigma_{\mathrm{PSN}}^2 + (c_{\mathrm{eff}} n \sigma_{\tilde{J}_z})^2} \tag{36}$$

$$= \sqrt{\left(\sqrt{2 n_1 m}\right)^2 + \left(c_{\mathrm{eff}} n_1 m \sqrt{\frac{1}{2N_{\mathrm{NV}}}}\right)^2}\ . \tag{37}$$

The experimental overhead is given by the time needed for the initialization of the nitrogen spin states (τ_{init}) and the nitrogen RF gate to map the electron spin onto the nitrogen spin (τ_{RF}) and the duration of the readout ($\tau_{\mathrm{r}} = m\tau_{\mathrm{r}}^{\mathrm{rep}}$) leading to a final expression of

$$\eta_{\mathrm{rep}} = \frac{1}{4\pi \gamma_e c \frac{m}{m_{T_1}}\left(1 - e^{-m/m_{T_1}}\right) n_1 \sqrt{\tau_{\mathrm{sens}}}} \sqrt{\frac{\tau_{\mathrm{sens}} + \tau_{\mathrm{init}} + \tau_{\mathrm{RF}} + m\tau_{\mathrm{r}}^{\mathrm{rep}}}{\tau_{\mathrm{sens}}}} \tag{38}$$

$$\sqrt{\left(\sqrt{2 n_1 m}\right)^2 + \left(2c\frac{m}{m_{T_1}}\left(1 - e^{-m/m_{T_1}}\right) n_1 m \sqrt{\frac{1}{2N_{\mathrm{NV}}}}\right)^2} \tag{39}$$

Since the sensitivity of the spin state readout using the repetitive readout scheme is limited by the spin projection noise, one possibility to improve it is to reduce the projection noise through spin squeezing. The squeezing parameter ξ^2 reduces the spin projection noise by $10^{-\xi}$, while increasing the experimental overhead by the time τ_{sq} to prepare the squeezed state. In this work, parameters for ξ^2 and τ_{sq} were taken from [14] A summary of the parameters used for the sensitivity calculation for Fig. 5 of the main text are given in Supplementary Tab. 3.

Supplementary Table 3: Parameters for sensitivity calculations.

τ_r [μs]	τ_r^{rep} [μs]	τ_{init} [μs]	τ_{RF} [μs]	τ_{sq} [μs]	n_1 [kcps]	c [%]	m_{T_1}
1.0	7.5	5236	600	3.0	0.036 N_{NV}	15	50000

Supplementary References

[1] R. Maier, C.-I. Ho, H. Sumiya, S. Onoda, J. Isoya, V. Vorobyov, and J. Wrachtrup, Efficient detection of statistical rf fields at high magnetic field with a quantum sensor (2025), arXiv:2503.12954 .

[2] P. Schätzle, P. Reinke, D. Herrling, A. Götze, L. Lindner, J. Jeske, L. Kirste, and P. Knittel, physica status solidi (a) **220**, 2200351 (2023).

[3] T. Miyazaki, Y. Miyamoto, T. Makino, H. Kato, S. Yamasaki, T. Fukui, Y. Doi, N. Tokuda, M. Hatano, and N. Mizuochi, Applied Physics Letters **105**, 261601 (2014).

[4] Y. Nakano, X. Zhang, K. Kobayashi, T. Matsumoto, T. Inokuma, S. Yamasaki, C. E. Nebel, and N. Tokuda, Diamond and Related Materials **125**, 108997 (2022).

[5] J. Michl, T. Teraji, S. Zaiser, I. Jakobi, G. Waldherr, F. Dolde, P. Neumann, M. W. Doherty, N. B. Manson, J. Isoya, and J. Wrachtrup, Applied Physics Letters **104**, 102407 (2014).

[6] E. Bauch, S. Singh, J. Lee, C. A. Hart, J. M. Schloss, M. J. Turner, J. F. Barry, L. M. Pham, N. Bar-Gill, S. F. Yelin, and R. L. Walsworth, Physical Review B **102**, 134210 (2020), publisher: American Physical Society.

[7] N. Aslam, M. Pfender, P. Neumann, R. Reuter, A. Zappe, F. Fávaro de Oliveira, A. Denisenko, H. Sumiya, S. Onoda, J. Isoya, and J. Wrachtrup, Science **357**, 67 (2017).

[8] V. V. Vorobyov, J. Meinel, H. Sumiya, S. Onoda, J. Isoya, O. Gulinsky, and J. Wrachtrup, Physical Review A **107**, 042212 (2023).

[9] C. L. Degen, F. Reinhard, and P. Cappellaro, Reviews of Modern Physics **89**, 10.1103/RevModPhys.89.035002 (2017).

[10] P. Neumann, J. Beck, M. Steiner, F. Rempp, H. Fedder, P. R. Hemmer, J. Wrachtrup, and F. Jelezko, Science **329**, 542 (2010).

[11] H. J. Carmichael, *Statistical Methods in Quantum Optics 2*, Theoretical and Mathematical Physics (Springer Berlin Heidelberg, Berlin, Heidelberg, 2008).

[12] J.-P. Amiet and S. Weigert, Journal of Optics B: Quantum and Semiclassical Optics **1**, L5 (1999).

[13] R. H. Dicke, Physical Review **93**, 99 (1954).

[14] W. Wu, E. J. Davis, L. B. Hughes, B. Ye, Z. Wang, D. Kufel, T. Ono, S. A. Meynell, M. Block, C. Liu, H. Yang, A. C. Bleszynski Jayich, and N. Y. Yao, Nature **646**, 74 (2025).
